# Cyanobacteria and Microalgae as Sources of Functional Foods to Improve Human General and Oral Health

**DOI:** 10.3390/molecules25215164

**Published:** 2020-11-06

**Authors:** Gianmaria Fabrizio Ferrazzano, Cristina Papa, Antonino Pollio, Aniello Ingenito, Giancarla Sangianantoni, Tiziana Cantile

**Affiliations:** 1 Department of Neurosciences, Reproductive Sciences and Oral Sciences, School of Paediatric Dentistry, University of Naples “Federico II”, 80131 Naples, Italy; cristina.papa95@gmail.com (C.P.); ingenito@unina.it (A.I.); giancarla.sangia@yahoo.com (G.S.); tizianacantile@yahoo.it (T.C.); 2 Unesco Chair on Health Education and Sustainable Development, University of Naples, “Federico II”, 80121 Naples, Italy; 3 Department of Biology, University of Naples “Federico II”, 80126 Naples, Italy; antonino.pollio@unina.it

**Keywords:** microalgae, *Spirulina platensis*, *Chlorella vulgaris*, oral health, antimicrobial activity, *Streptococcus mutans*

## Abstract

In the scenario of promising sources of functional foods and preventive drugs, microalgae and cyanobacteria are attracting global attention. In this review, the current and future role of microalgae as natural sources of functional foods for human health and, in particular, for oral health has been reported and discussed in order to provide an overview on the state of art on microalgal effects on human oral health. It is well known that due to their richness in high-valuable products, microalgae offer good anti-inflammatory, antioxidant, antitumoral, anti-glycemic, cholesterol-lowering, and antimicrobial activity. Moreover, the findings of the present research show that microalgae could also have a significant impact on oral health: several studies agree on the potential application of microalgae for oral cancer prevention as well as for the treatment of chronic periodontitis and different oral diseases with microbial origin. Thus, beneficial effects of microalgae could be implemented in different medical fields. Microalgae and cyanobacteria could represent a potential natural alternative to antibiotic, antiviral, or antimycotic therapies, as well as a good supplement for the prevention and co-adjuvant treatment of different oral diseases. Nevertheless, more studies are required to identify strains of interest, increase overall functioning, and make safe, effective products available for the whole population.

## 1. Introduction

Today, the majority of the bioactive peptides added in well-being promoting aliments, food supplements, pharmaceutic, and cosmetic formulations are realized by chemical production or by the partial digestion of proteins derived from animals. Therefore, they are not always well-regarded by the users due to the risks related to solvent contamination or the utilization of animal-derived ingredients. Alternatively, plant and microalgae-resulting peptides are recognized as selective, useful, nontoxic and well accepted when consumed, having a significant potentiality for utilization in functional aliments, drugs, and cosmetic products. Furthermore, due to the increasing issue of the antibiotic resistance against pathogenic bacteria, research has been targeted to exploring new antibacterial compounds derived from different natural environments [1,2,3,4]. Consequently, in recent years, growing scientific interest has been centered on the study of the bioactivity of extracts derived from plant species. In particular, the focus is on food plants included in the so-called traditional medicine [5,6,7,8]. For example, polyphenols derived from some edible plants drew attention as potential sources of agents that, among the wide range of health benefits, were shown to inhibit the bacterial growth of some oral pathogens [9,10]. A recent study on a large sample of vulnerary plants from Italian areas has identified a small number of extracts that could find application for the prevention of dental caries [11]: extracts of *Camelia sinensis* and *Plantago lanceolata*, rich in flavonoids, showed inhibitory activity against the species of cariogenic Streptococci [11,12].

Functional foods are arising as a dietary resource for preventing diseases. They can be defined as technologically developed ingredients explicating specific actions on human health. In the scenario of growing promising sources of functional foods and preventive drugs, cyanobacteria and microalgae are drawing global interest, due to their content of highly valuable substances [13,14].

Cyanobacteria and microalgae are respectively prokaryotic or eukaryotic microorganisms growing through oxygenic photosynthesis. Their energetic intake requires light, carbon dioxide, water, and nutrients with phosphorus and nitrogen as major nutrients, whereas the products of metabolic activity are primarily carbohydrates, along with a surprisingly high number of chemicals, some of which are known to be useful for humans [13].

**Figure 1:**
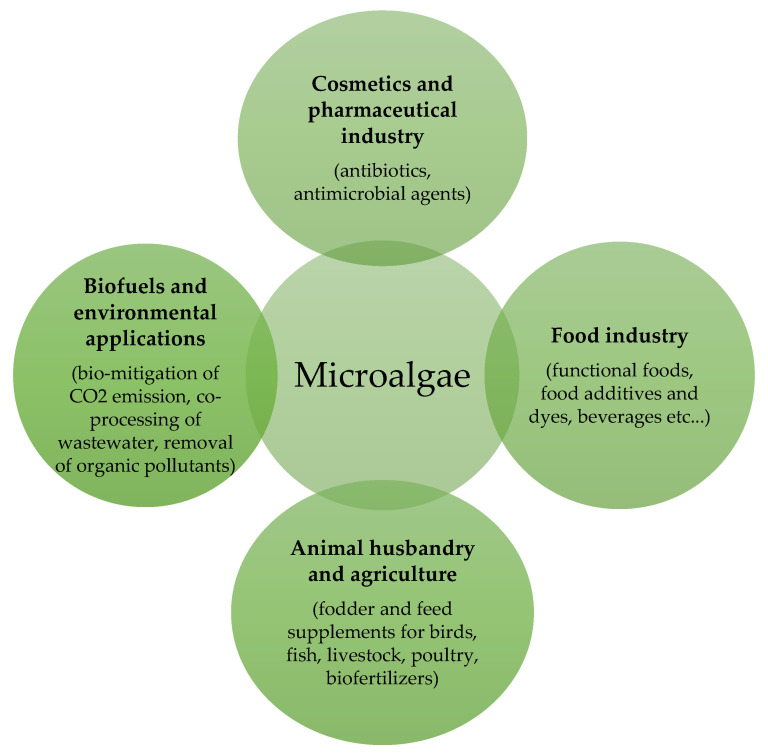
Application of microalgae in different fields.

Both cyanobacteria and microalgae are widely spread in freshwater and marine ecosystems as well as on terrestrial habitats and in a wide range of extreme environments, from hot springs to barren rocks of deserts. This flexibility makes them the major producers of biomass under sustainable conditions, since they do not deprive agriculture of sources, while allowing water recycling and lowering polluting gas emissions [15]. Moreover, cyanobacteria and microalgae bring the potential to be converted into a wide range of products of economic interest, such as biofuels, cosmetics, renewable chemicals and other valuable compounds [15], animal and human food, especially as sources of proteins, lipids, and phytochemicals (Figure 1) [16].

In this review, the present and future role of cyanobacteria and microalgae as sources of drugs and food supplements useful for human well-being and, in particular, for oral health has been reported and discussed, focusing on the main classes of microalgal compounds with health implications. The aim is to offer a view on the state of art on the microalgal role and potential benefits to human well-being and, in particular, to oral health.

## 2. Health Beneficial Effects of Cyanobacteria and Microalgae Crude Extracts and Fractions

### 2.1. A Taxonomic Controversy: Arthrospira vs. Spirulina

*Spirulina platensis* and *Spirulina maxima* are the most frequently utilized names to designate the strains of filamentous cyanobacteria worldwide cultivated as food or to produce fine chemicals. However, both names are incorrect from a taxonomical point of view and have generated confusion on the identification of the most promising strains. The genera *Spirulina* and *Arthrospira* were erected respectively by Turpin in 1829 and Stitzenberger in 1852; this latter author proposed including in the new genus *Arthrospira* helical-shaped Oscillatoriaceae, which were previously included in *Spirulina*. Since then, the two genera have been reunited and separated several times, until in 1989, Castenholtz proposed distinguishing *Arthrospira* from *Spirulina* based on selected morphological features such as a degree of inclination of the trichome axis, the presence of cross walls in the filament, and the cell wall pore pattern. Molecular analysis confirmed the separation of the two genera and evidenced difficulties in the circumscription of the species generally accepted [17]. However, most of the strains worldwide cultivated belong to the genus *Arthrospira*, even though they are commonly sold as *Spirulina*. Unfortunately, also, many studies refer to *Arthrospira* strains as “*Spirulina*”, engendering further confusion. To avoid misunderstandings, in this review, we have maintained the names adopted by the authors of each study independently of their taxonomical consistency.

With its high nutritional value, *Arthrospira*/*Spirulina* has been consumed as food for centuries in Central Africa [18]. Thanks to its high content of important proteins, amino acids, vitamins, carotenoids and other pigments, mineral substances, indispensable fatty acids, and polysaccharides, *S. plantensis* has found application as bioactive additive, and it is now widely used as a nutraceutical food supplement worldwide [19].

*S. platensis* causes an immunostimulating action, improving the resistance of humans, mammals, chickens, and fish to infections, thanks to the ability of affecting hemopoiesis, stimulating the production of antibodies and cytokines [20]. Under the effect of *S. platensis*, macrophages, T and B cells are activated. *S. platensis* sulfolipids have shown to be valid against HIV. Moreover, *S. platensis* biomass supplementations have appeared to be effective against herpes virus, cytomegalovirus, influenza virus, etc. *S. platensis* extracts are capable of impeding cancerogenesis [18] (Table 1).

*S. platensis* preparations are known as functional products playing a role in preserving the resident intestinal microflora, principally lactic acid bacilli and bifidobacteria, and in decreasing the level of *Candida albicans* [18]. In particular, in 2017, in vitro experiments assessed the impact of a fully characterized water extract of *S. platensis* against 22 varieties of *Candida* spp. The natural waves pattern of uterine myometrium and the major health-promoting bacteria of the vaginal flora were not affected by *S. platensis*, which was administered at the concentrations active against *Candida* [21].

*S. platensis* stimulates cellular antioxidant enzymes, reduces lipid peroxidation and DNA damage, scavenges free radicals, and increases the effect of superoxide dismutase and catalase. According to clinical studies conducted in 2016, *S. platensis* protects skeletal muscle from injury under exercise-generated oxidative stress and can induce the production of antibodies and up- or downregulate the expression of cytokine-encoding genes involved in the immunomodulatory and anti-inflammatory processes. Although the molecular mechanism(s) by which *S. platensis* makes these actions is still partially unknown, phycocyanin and β-carotene are likely to give a crucial contribution [22].

The Paracas strain of *S. platensis* is renowned as a potential source of precious skin-beneficial substances and recently, it has been applied in skincare products. Gunes et al. (2017) developed and analyzed skin creams enriched with bioactive *S. platensis Paracas* extract. Collagen 1 immunoreactivity was notably ameliorated along with the increase of extract concentration, as demonstrated by immunohistochemical analysis. The cell viability, wound curative action, and genotoxicity results indicated that *S. platensis* strain *Paracas*-enriched skin cream can bring highly valuable benefits in cosmeceutical and biomedical applications [23].

Eventually, it is worth mentioning the case study conducted by Kiziltan et al. in 2015, intending to evaluate the advantages of combining therapy with *S. platensis* and metronidazole with radiotherapy to ameliorate the treatment response of relapsed verrucous vulvar cancer. After the adjunction of conventional therapy with microalgae assumption, the tumor regressed, and there were no adverse effects involving the woman’s dermatological or general health [24].

### 2.2. Chlorella sp.: A Multifunctional Dietary Supplement with Diverse Medical Properties

*Chlorella* is a unicellular microalgae genus belonging to the class Treouxiophyceae (Chlorophyta) with biological and pharmacological potential important for human well-being. “*Chlorella* growth factor”, the hot water extract of *Chlorella*, appeared to be active in the growth promotion and immunoregulation [25]. At the present moment, no systematic analysis has assessed the state of research of *Chlorella* sp. and its bioactivity. However, according to the growing scientific literature, *Chlorella* sp. not only has great importance in immunoregulation and cancer inhibition, but it is also effective in ameliorating metabolic syndrome, scavenging for free radicals, protecting from ultraviolet damage, chelating heavy metals, and preventing liver and bowel disease [25]. Nevertheless, not all scientific literature is consistent with these findings: for example, a randomized, double-blind, placebo-controlled trial in 2003 showed that *Chlorella*-derived dietary supplement did not exhibit any action in increasing the antibody response to influenza vaccine in the whole study population [26].

According to clinical studies conducted in 2016, integration with *Chlorella vulgaris* can ameliorate hyperlipidemia and hyperglycemia and protect from oxidative stress, cancer, and chronic obstructive pulmonary disease [27]. In 2013, Panahi et al. investigated the effect of supplementation with *C. vulgaris* on the burden of oxidative stress in Iranian smokers. After six weeks of supplementation, this integrator significantly enhanced the antioxidant status and reduced lipid peroxidation in chronic cigarette smokers. Therefore, *C. vulgaris* could impede the disease burden and mortality rate connected with smoking [28]. In another study carried out in 2015, the same author assessed the medicinal beneficial effect of an antioxidant-rich *C. vulgaris* ethanolic extract administered as a supplement of standard therapy in subjects with major depressive disorder (MDD), as oxidative stress has been reported to contribute significantly in the pathophysiology of MDD. This pilot study gives the first scientific evidence on the effectiveness and safety of supplementary therapy with *C. vulgaris* ethanolic extract in enhancing physical and cognitive symptoms of depression and anxiety in subjects undergoing standard antidepressant treatment [29].

In 2017, an interventional trial studied the consequences of *C. vulgaris* supplementation on glucose homeostasis, insulin resistance, and inflammatory biomarkers in subjects with non-alcoholic fatty liver disease [30]. This condition, representing a hepatic symptom of metabolic syndrome, is highly related to insulin resistance and inflammation. Weight decrease (mean values) was significantly higher in the *C. vulgaris*-treated group with respect to the placebo group. Serum concentrations of liver enzymes also significantly diminished, and serum insulin concentration significantly improved only in the group that had undergone *C. vulgaris* therapy. According to these findings, *C. vulgaris* supplementation could be considered as an adjunctive therapy to reduce weight and improve glycemic status as well as to ameliorate liver function in subjects with non-alcoholic fatty liver disease [30].

An in vitro study in 2016 compared the antibacterial activity of the methanolic extracts of five cyanobacteria and microalgal species, namely *Nostoc caeruleum*, *S. platensis*, *Cylindrospermum majus*, *Oscillatoria formosa*, and *C. vulgaris* against six bacteria (*Staphylococcus aureus*, *Staphylococcus epidermidis*, *Streptococcus pyogenes*, *Klebsiella pneumoniae*, *Pseudomonas aeruginosa,* and *Escherichia coli*) and four fungi strains (*Aspergillus fumigatus*, *C. albicans*, *Geotrichum candidum,* and *Trichophyton mentagrophytes*). The results suggested that the extract of *C. vulgaris* was the most effective agent against the tested bacteria and fungi strains, followed by *S. platensis* [31]. Other studies, conducted respectively in 1988 and 2015, agree that antibacterial and antimicotic effects are prevalent from *Chlorella* sp. [32,33]. Furthermore, according to chemical analyses, *C. vulgaris* showed the highest percentages of total phenolic and total flavonoid contents, which are identified as mainly responsible for antioxidant potential. *C. vulgaris* extract was also tested for its antitumor activity against specific cancer cell lines: the obtained data indicate that liver cancer cells HepG-2 were the most sensitive, followed by colon cancer cells HCT-116, and then breast cancer cells MCF-7 [25].

In 2017, Lin et al. studied for the first time the anti-cancer properties of *Chlorella sorokiniana* in two human non-small lung cancer cell lines (A549 and CL1–5 human lung adenocarcinoma cells), and its action on cancer growth in a subcutaneous xenograft tumor model. The results proved that exposure of the two cell lines to *C. sorokiniana* determined a concentration-dependent decrease in cell viability and a dose-dependent increase in the number of apoptotic cells. In addition, it was detected that the tumor growth of the subcutaneous xenograft in vivo was notably reduced after oral administration of *C. sorokiniana* [34].

These promising results suggest that *Chlorella* sp. extracts could represent a source of antitumor, anti-inflammatory, antioxidant, and antimicrobial bioactive compounds (Table 2). However, more studies are needed to investigate all the clinical effects of *Chlorella*-derived dietary supplements on human health.

## 3. Microalgal Effects on Oral Health

In the current scientific literature, there is still a lack of evidence about benefits derived from microalgal use in terms of treatment and the prevention of oral diseases. The collected studies mainly claim the potential role of *Spirulina* sp. in improving oral health.

### 3.1. Antiviral Activity

Herpes virus is a double-stranded DNA virus from the family of Herpesviridae. As its peculiarity, this virus does not leave the host after the first contact, settling in neural ganglion and causing a latent infection. This can take place in a variable time, depending on the type of virus and the host susceptibility. The causes that induce the reactivation of the viral activity can be temperature changes (hot/cold), traumas, fever, stress, and, above all, reduction of the host immunological response [35].

A study carried out in 2016 examined the antiviral activity of a defined *S. platensis* microalgal extract and of purified calcium spirulan (Ca-SP), which is a sulfated polysaccharide contained therein. Ca-SP inhibited HSV-1 infection in vitro with an effectiveness similar to that of acyclovir by blocking viral attachment and penetration into host cells. Ca-SP also impeded the entry of Kaposi sarcoma-associated herpesvirus/human herpes virus 8. Actually, in the experimental model of herpes exacerbation, the prophylactic activity of a cream containing Ca-SP and microalgae extract was greater than that of acyclovir cream. These results highlight a possible clinical use of Ca-SP containing *Spirulina* sp. extract for the prophylactic management of *herpes labialis* and indicate promising effects of Ca-SP against infections caused by other herpes viruses [36].

### 3.2. Oral Cancer Chemoprevention

Another interesting application for *Spirulina* sp. and, in particular, for the blue-green microalga *S. platensis* might be in oral cancer chemoprevention. Oral cancer represents 3% of all human carcinomas. Squamous cell carcinoma accounts for over 90% of the oral cancers, and others include adenocarcinoma derived from minor salivary gland, sarcoma, malignant lymphoma, and metastatic cancer. It shows a survival rate at 5 years of 51%, which is mainly due to late diagnosis in a clinical advanced stage. It is localized most frequently on the tongue, on the floor of the mouth, and on the cheek mucous, and it is mainly related to alcohol, smoking, and Human Papilloma Virus (HPV) infection [37]. Especially, smoke-related cancer is often preceded by some typical preneoplastic lesions, such as oral leucoplakia, erytroplakia, lichen planus, or persistent ulcers. Today, conventional therapy of potential pre-neoplastic conditions is mostly limited to mouth washes with antibacterial and antimycotic products in order to remove any superinfections, along with the elimination of risk factors, patient follow-up, and eventually, biopsy. On the other hand, the first choice for oral cancer treatment is still the surgical approach, causing inevitable esthetic and functional impairments [38]. Hence, there is a huge need to develop specific and less invasive strategies for oral cancer prevention and therapy.

Mathew et al. in 1995 evaluated the chemopreventive activity of *Spirulina fusiformis* (1 g/day for 12 months) in reversing oral leukoplakia in pan tobacco chewers in Kerala, India. Complete regression of lesions was documented in 20 of 44 (45%) evaluable patients supplemented with *S. fusiformis*, as opposed to three of 43 (7%) in the placebo arm. When stratified by type of leukoplakia, the response was more significant in homogeneous lesions: complete regression was observed in 16 of 28 (57%) patients with homogeneous leukoplakia, two of eight with erythroplakia, two of four with verrucous leukoplakia, and zero of four with ulcerated and nodular lesions. During one year of discontinuing supplements, nine of 20 (45%) complete responders with *S. fusiformis* developed recurrent lesions. Supplementation with *S. fusiformis* did not provoke any adverse effect [39]. This was the first human study assessing the chemopreventive potential of *S. fusiformis*.

Another study conducted in 2015 estimated if astaxanthin could be applied to ameliorate the symptoms of oral lichen planus by decreasing inflammation. Following astaxanthin treatment, the level of inflammatory cytokines (interleukin-6, tumor necrosis factor-α) tended to decrease, and cell proliferation significantly increased in human gingival keratinocytes in vitro. These results indicate that astaxanthin could be beneficial for improving chronic inflammation such as that associated with oral lichen planus [40].

Several experimental studies in animal models have demonstrated an inhibitory effect of *S. platensis* microalgae on oral carcinogenesis process. Some studies conducted on rats sustain that astaxanthin has significant preventive activities on 4-nitroquinoline-1-oxide (4-NQO)-induced tongue carcinogenesis [41]. In particular, after 8 weeks of treatment, the incidence of oral preneoplastic lesions and cell proliferation activity decreased significantly, and no oral neoplasms developed in rats fed with astaxanthin [42]. Grawish and his team carried out two experimental studies on hamsters (2008 and 2010) both aiming to investigate oral chemopreventive strategies using astaxanthin. In the first work, 30 male golden Syrian hamsters were divided into three groups: the first group had the right buccal pouch painted with 0.5% solution of 7,12-dimethylbenz[a]anthracene (DMBA), to the second group, 10 mg/daily *S. platensis* extract was administered, in addition to the same painting, and the last was a control group, not having painting nor *S. platensis* treatment. They observed moderate dysplastic changes extending into the midspinous layer in group one 7 weeks after DMBA painting, which reached half the thickness of the hyperplastic epithelium after 14 weeks. However, in group two, mild dysplastic changes were observed after 7 weeks, which were restricted to the basilar and parabasilar layers of the epithelium after 14 weeks of treatment. An overall significant difference among the three groups (*p* < 0.01) was indicated with one-way analysis of variance [43]. The second study from the same author confirmed these results. In particular, it focused on the proliferating cell nuclear antigen expression, showing that it was directly related to the severity of pathological alterations from normal epithelium to dysplasia and from dysplasia to squamous cell carcinoma in the study groups at the different extended periods of DMBA application and *S. platensis* extract administration [44].

*S. platensis* extracts, especially astaxanthin carotenoid, might have a big potential not only on precancerous lesions, but even on proper oral cancer. As in 1988, it was already proven that an extract of *S. platensis*–*Dunaliella* could inhibit tumor development in hamster buccal pouch [45], more recent data provided extensive evidence that dietary astaxanthin avoids the development and progression of hamster buccal pouch carcinomas through the inhibition of JAK-2/STAT-3 signaling and its downstream events [46].

Many scientists investigated the mechanisms behind *S. platensis* potent anti-cancer activity. Their results are summarized as follows.

In 2019, Sannasimuthu et al. identified an RNA sequence encoding the glutathione oxido-reductase (GR) enzyme from *S. platensis*, and the modifications in its gene expression profile were investigated throughout H_2_O_2_ stress. GR is a primary antioxidant enzyme of many organisms that prevents cellular oxidative stress by reducing glutathione from its oxidized form. Overall, the study demonstrates that the GM15 peptide (a short antioxidant peptide from *S. platensis* glutathione oxido-reductase) scavenges superoxide, hydroxyl radicals, reduces intracellular oxidative stress, and has antitumor activity in oral cancer cells [47]. According to Kavitha et al. (2013), astaxanthin exerts chemopreventive effects by concurrently inhibiting the phosphorylation of transcription factors and signaling kinases, and inducing intrinsic apoptosis. In fact, they found that the microalgal carotenoid inhibits NF-κB (nuclear factor kappa-light-chain-enhancer of activated B cells) and Wnt (wingless/integrated) signaling by downregulating the key regulatory enzymes IKKβ and GSK-3β [48]. Eventually, in a recent investigation, Kowshik et al. (2019) reported that astaxanthin prevents cancer hallmarks by inhibiting PI3K/Akt and the associated downstream NF-κB and STAT-3 signaling pathways in SCC131 and SCC4 oral cancer cells as well as in the hamster buccal pouch carcinogenesis model. Additionally, astaxanthin downregulated the noncoding RNAs (ncRNAs), miR-21 and HOTAIR, which influence PI3K/Akt signaling, emphasizing their modulatory effects on epigenetic regulation [49].

Taking into account all these promising results, it can be concluded that *S. platensis* and, particularly, its most potent pigment, astaxanthin, plays a beneficial role in oral cancer regression and prevention, representing a potential valuable candidate for anti-cancer drug development.

### 3.3. Oral Antimicrobial Activity

Another property of cyanobacteria and microalgae that appears to be worthy of note for oral health implications is their antimicrobial activity. Among oral bacteria, *Streptococcus mutans* is a Gram-positive bacteria that is frequently found in the oral cavity’s normal flora and involved in the pathogenesis of caries [50,51]. Caries is the most common infective pathology worldwide, affecting especially that part of the population with a lower socio-economic status. It has a multifactorial pathogenesis, including diet and host susceptibility as co-factors together with the over-growing of *S. mutans*. *S. aureus* is also an abundant Gram-positive bacterium that has a strong connection with dental implant infections [52]. *Enterococcus faecalis* has been connected to oral infections, such as caries, endodontic infections, periodontitis, and peri-implantitis [53].

Fatty acid methyl esters (FAME) derived from lipids of microalgae are recognized as a suitable super-curator and superior anti-pathogenic. Experiments in 2018 assessed the effectiveness of FAME extracted from the microalga *Scenedesmus intermedius* as an antimicrobial agent against Gram-positive bacteria (*S. aureus*, *S. mutans*, and *Bacillus cereus*), Gram-negative bacteria (*E. coli* and *P. aeruginosa*), and fungi (*Aspergillus parasiticus* and *C. albicans*). FAME here analyzed displayed a solid antimicrobial effect at a lower MIC than those of recent reports. This result claimed that the FAME of *S. intermedius* has a potent antimicrobial and antioxidant activity and that it could find application as a powerful source against microbial diseases [54].

Microalgal extracts screened for their antimicrobial activities could be more effective than antibiotics and fungicides. An in vitro study in 2011 demonstrated the antimicrobial properties of *Chaetoceros calcitrans*, *Skeletonema costatum*, *Chroococcus turgidus,* and *Nannochloropsis oceanica*. Extracts exhibited inhibitory effects against *S. aureus*, *S. pyogenes,* and *Bacillus subtilis*. Antifungal activity was identified only in *S. costatum* and *C. turgidus*, but *Aspergillus flavus* and *A*. *niger* showed resistance to all the four microalgae. The highest in vitro inhibition zone was observed in acetone extract of *Chlorococcum* sp. against *S. aureus* and *E. coli* compared to other dried green microalgae extracts [55].

Another in vitro study in 2011 collected 46 species of freshwater cyanobacteria and selected five strains based on their growth characteristics, namely *Oscillatoria latevirens*, *Phormidium corium*, *Lyngbya martensiana*, *Chrococcus minor,* and *Microcystis aeruginosa*. The results clearly indicated that the acetone extracts of *O. latevirens* and ethanol extracts of *P. corium* gave the highest antimicrobial activity against *S. aureus*, *S. mutans,* and *Micrococcus mutans* and *S. aureus*, respectively. At the same time, the extract of *L. martensiana* had an antibacterial effect toward *B. subtilis*, *S. aureus,* and *E. coli* but negative effect toward *S. mutans*, *M. mutans*, and *K. pneumoniae*. In addition, *O. latevirens*, *C. minor,* and *M. aeruginosa* were shown to have antifungal activity on *C. albicans*. It was found that the effect of standard antibiotics was more than that of algal extracts on *B. subtilis* and *E. coli*. Meanwhile, the antibacterial potential of algal extracts on *S. aureus*, *S. mutans,* and *M. mutans* were even higher than those of standard antibiotics. The antimicrobial activity of these microalgae could be explained by presence of cyclic peptides, alkaloids, and lipopolysaccharides [56].

An investigation carried out in 2016 used *S. platensis* extract as a biomaterial for the biosynthesis of silver nanoparticles. Nanoparticles have unique biological and optical properties, which make them considered as efficient materials of the next technology generation with therapeutic and diagnostic applications. Metal nanoparticles are highly employed in a broad number of biomedical applications, especially as antimicrobials [57]. Silver ions and silver-based compounds are known bactericides, due to their large surface area that comes into contact with the microbial cells [58,59]. The application of nanoparticles in dentistry is of interest, as the oral cavity frequently comes across a superfluity of microorganisms. The results of the study suggest that the silver nanoparticles biosynthesized using *S. platensis* extract have good antibacterial activity against the three Gram-positive oral pathogens *S. mutans*, *E. faecalis,* and *S. aureus* [60].

### 3.4. Potential Effects in the Treatment of Periodontitis

Oral periodontitis is an inflammatory pathology affecting the supporting gum and bone tissues of the tooth. It is characterized by a cyclical evolution, alternating phases of quiescence with acute phases, which determines an irreversible loss of the clinical epithelium-connective attachment around the tooth and, as a consequence, progressive bone resorption [61]. Today, chronic periodontitis mostly requires three conventional treatment modalities: non-surgical techniques as scaling and root plaining, surgical conservative approaches for more compromised teeth and, ultimately, the extraction of hopeless teeth [62].

Several studies claim the potential clinical effectiveness of *S. platensis* for the treatment of oral periodontitis, which is probably due to its anti-inflammatory and antioxidant effects. Mahendra et al. in 2013 demonstrated that locally delivered *S. platensis* gel, along with scaling and root plaining, caused a beneficial impact in the treatment of chronic periodontitis. The efficacy of the product as a local drug delivery system in the non-surgical management of periodontitis without any side effects has been tested [63]. According to these findings, a recent pre-clinical study evaluated the effects of astaxanthin on alveolar bone loss and histopathological changes in ligature-induced periodontitis in rats. It can be suggested that astaxanthin administration may reduce alveolar bone loss by enhancing osteoblastic activity and decreasing osteoclastic action in an experimental periodontitis model [64]. The suppression effect of astaxanthin on bone loss is confirmed by Hwang et al. (2018), who studied the anti-osteoporotic effect of astaxanthin on bone mass in ovariectomized mice and its possible mechanism of action. The administration of astaxanthin (5, 10 mg/kg) for six weeks suppressed the enhancement of serum calcium, inorganic phosphorus, alkaline phosphatase, total cholesterol, and tartrate-resistant acid phosphatase activity. The bone mineral density and bone microarchitecture were improved by astaxanthin exposure [65].

Furthermore, in 2016, a study evaluated the effects of another microalgal carotenoid, fucoxanthin, on alveolar bone resorption in rats with periodontitis. Systemic fucoxanthin treatment resulted in a small reduction in tumor necrosis factor-α, interleukin-1β, and interleukin-6 levels and a significant reduction in oxidative stress index. It was noted that fucoxanthin induced a significant decrease in receptor activator of nuclear factor kappa-β ligand (RANKL) levels and a statistically non-significant elevation in osteoprotegerin and bone–alkaline phosphatase levels. There were no significant differences in alveolar bone loss levels between the case and control groups. Hence, according to this experimental study, fucoxanthin determines a limited decrease in alveolar bone resorption in rats with periodontitis [66].

Eventually, a recent work in 2018 analyzed the effects of a cyanobacterial component on the inflammation process induced by *Phorphyromonas gingivalis*, which is a Gram-negative bacterium co-responsible for oral periodontitis. Lipopolysaccharide (LPS) from *P. gingivalis* (Pg-LPS) is a key bacterial structure involved in the maintenance of a chronic pro-inflammatory environment during periodontitis. Similar to other Gram-negative LPS, Pg-LPS induces the release of pro-inflammatory cytokines through interaction with Toll-Like Receptor 4 (TLR4) and is able to stimulate negative TLR4 regulatory pathways, such as those involving microRNA (miRNA). In this work, the authors employed CyP, an LPS with TLR4-MD2 antagonist activity obtained from the cyanobacterium *Oscillatoria planktothrix* FP1, to study the effects on pro-inflammatory cytokine production and miRNA expression in human monocytic THP-1 cells stimulated with Pg-LPS. The results showed that CyP inhibited TNF-α, IL-1β, and IL-8 expression [67]. These outcomes could open new perspectives for innovative therapeutic approaches based on microalgal and cyanobacterial extracts for the treatment of oral periodontitis.

### 3.5. Control of Oral Submucous Fibrosis

A new horizon in *S. platensis* use can be in the management of an insidious and disabling disease affecting oral mucosa, such as Oral Submucous Fibrosis (OSMF). OSMF is a chronic progressive pathology that alters the flexibility of oral mucosa, ending in lockjaw and a higher risk of developing squamous oral cancer. It is a common condition in India and southeastern Asia, while it is infrequent in other parts of the world [68]. The results of trials of many treatment options, such as medical, physical, or surgical interventions, have suggested that combined therapies give better outcomes. A study conducted in 2019 intended to assess the effectiveness of *S. platensis* together with different physiotherapeutic approaches in the treatment of OSMF. After administration of *S. platensis* 500 mg twice a day for 3 months, all subjects have reported statistically significant amelioration in burning perception, mouth opening, tongue protrusion, and cheek flexibility. Moreover, *S. platensis* did not cause any considerable adverse effect [69]. Another experimental work carried out in 2013 evaluated the efficacy of the same *S. platensis* treatment in addition to corticosteroid injections in the treatment of oral submucous fibrosis symptoms. After 3 months of therapy, highly significant clinical improvements in mouth opening and reduction in burning perception were recorded in favor of the *S. platensis* group [70]. The reported effects indicate that *S. platensis* could be applied as an adjuvant therapy in the treatment of initial symptoms in subjects with OSF. Nevertheless, studies involving larger samples and longer follow-up are recommended in the future [71].

### 3.6. Salivary Secretion Improving

Salivary secretion is fundamental for the oral well-being, considering that hyposalivation compromises food chewing and swallowing, reduces mucosal immune function, increases the risk for oral diseases such as dental caries and candidiasis, and causes dysphagia, dysgeusia, and prosthetical inadaptation. Oral dryness is normally associated to aging or menopause, but it can also be caused by some pharmacological therapies, radio- and chemotherapy, or Sjogren’s syndrome. In any case, oxidative stress clearly plays a role in decreasing saliva secretion. Then, treatment with antioxidant agents, such as astaxanthin, may be beneficial [72].

Kuraji et al. (2015) estimated the effects of astaxanthin on the saliva secretory function of aging mice. The saliva flow increased in the case group 72 weeks after treatment, while that of the control decreased by half. Moreover, the submandibular glands of astaxanthin-treated mice had fewer inflammatory cells than the control did [72]. These results were corroborated by the work carried out by Yamada et al. (2010), which was structured in three parts. First, they evaluated the reactive oxygen species (ROS) scavenging capacity of astaxanthin on a human salivary gland epithelial cell line and obtained that the carotenoid partially suppressed hydrogen peroxide-induced ROS in salivary gland cells. Secondly, they examined the effects of astaxanthin on salivary secretion in a mouse model of irradiation-induced salivary gland dysfunction, resulting in the inhibition of oral dryness. Lastly, they tested astaxanthin treatment in six patients affected by Sjogren’s syndrome compared to a control group: an increase in salivary flow was detected in both groups of patients, and the level of oxidative stress marker, hexanoyil-lysine, in the saliva was decreased after astaxanthin administration [73].

According to Otsuki et al. (2016), *Chlorella* sp.-derived multicomponent supplementation, as well, is able to increase saliva production in subjects with lower levels of saliva secretion [74].

These results suggest that therapy with extracts derived from *Spirulina* sp. or *Chlorella* sp. has a good potential for the prevention and treatment of different clinical conditions of hyposalivation.

### 3.7. More Oral Benefits from Chlorella sp. and Other Algal Extracts

The long-term microalgal extract supplementation can bring several unexpected benefits to oral health.

*C. vulgaris* extract in conjunction with aminosulphurate (nutraceuticals) supplementation has determined a detoxification from heavy metals in subjects with long-term titanium dental implants and/or amalgam fillings compared to baseline levels and untreated controls. It is known how the accumulation of heavy metals represents a serious risk for human health. In particular, mercury toxicity can end in kidney dysfunction and neurologic anomalies. The administration of microalgae and nutraceutical supplements for 90 consecutive days reduced Hg^2+^, Ag, Sn, and Pb with respect to baseline levels and did not have any side effects [75].

Finally, another interesting application for microalgal-derived components might be as drug carriers for bisphosphonates. Bisphosphonates are a class of drugs widely used in the clinical treatment of disorders of bone metabolism, such as osteoporosis, fibrous dysplasia, myeloma, and bone metastases. As a result of the negative side effects caused by the oral administration of bisphosphonates, various silica mesoporous materials have been investigated for a confined and controlled release of these drugs. Following a novel strategy, sodium alendronate can be in vivo incorporated into biosilica shells of the diatom *Thalassiosira weissflogii* by feeding the algae with an aqueous solution of the drug. According to a recent study conducted in 2019, the loading percentage of sodium alendronate into biosilica was able to decrease the metabolic activity of J774 osteoclasts-like cells until 5% over glass control. Osteoclasts inhibition of the functionalized biosilica opens the way to interesting applications for diatom microalgae as a bioinspired mesoporous material for tissue engineering [76].

A summary of the main sources of bioactive compounds reviewed in the current study, bringing benefits for oral health, is provided in the following table (Table 3).

## 4. Conclusions

Cyanobacteria and microalgae are attracting global attention as promising sources of functional foods and preventive drugs, because of their richness in high-valuable products, comprising carotenoids, proteins, vitamins, essential amino acids, and omega-rich oils. They represent an almost untapped resource of natural anti-inflammatory and antioxidant compounds due to their enormous biodiversity, and a growing number of experiments suggests that the positive effects of microalgae on human well-being could be extended to the prevention or delay of cancer and cardiovascular diseases, as well as the management of diabetes, hypercholesterolemia, and inflammatory chronic diseases. Shifting the focus to oral health, several studies agree on the potential application of microalgae in the field of oral cancer prevention, in the treatment of a disabling condition as Oral Submucosal Fibrosis, and the management of chronic periodontitis complications. Moreover, due to their antiviral, antibacterial, and antifungal properties, microalgae could have a significant impact on oral health, acting as protective agents against *H. labialis*, *S. mutans,* and *C. albicans*.

Probably, one of the major constraints to a larger use of algal extracts and products is due to the erratic use of botanic nomenclature to designate the strains considered promising for biotechnological applications. Taking into account the two most studied and commercialized phototrophic microorganisms, *Arthrospira*/*Spirulina platensis* and *Chlorella*, a nomenclatural reassessment of the most frequently used strains is required, in light of the profound taxonomic changes that both genera have undergone in the last decade. This is the first important step needed in order to identify new strains of clinical interest, increase overall functioning, and make safe, effective products available for the whole population.

Cyanobacteria and microalgae cultivation can allow a high rate of biomass production, also achieving the synthesis of fine chemicals, as protein, carbohydrates, lipids, and pigments, that are used as components of human and animal feeds, biofuels, pharmaceuticals, and nutraceuticals. Moreover, phototrophic microorganisms can be grown on soils not suitable for agriculture, also using wastewater as a source of water. However, these promising economical perspectives are hindered by several difficulties; in particular, the need for large-scale facilities requires relevant investments that are not balanced by adequate revenues. At the moment, cyanobacteria and microalgae can be successfully grown in different kinds of bioreactors, but only open ponds are economically convenient. Therefore, in temperate zones, phototrophic microorganisms must face severe climatic conditions related to fall and winter seasons, with a consequent dramatic drop of production. There is an urgent necessity of identifying strains that are able to endure these constraints, ensuring adequate growth rates.

The possibility of using cyanobacteria and microalgae in the health market is appealing, but more stringent data are necessary to expand this possibility. The bottleneck is the scaling up of cultivation from laboratory-controlled conditions to the industrial production: the ability of strains to resist predation pressures and to co-grow with other microorganisms, such as bacteria, is a key aspect. In addition, the presence of both predators and commensal prokaryotes can induce relevant physiologic changes, leading to a decrease of fine chemical production and/or a shift toward the synthesis of unwanted metabolites. Experiments designed to overwhelm these constraints should focus on innovative biotechnology approaches, based on the activation or deactivation of specific genes to attain higher biomass production and synthesis of bioactive compounds [77].

## Figures and Tables

**Table 1:** Different actions of *S. platensis* on human health and related bibliographical references.

*S. platensis’s* Medicinal Properties	Ref.
Highly nutrient	[19]
Immunostimulating	[20]
Antiviral	[18]
Antimicotic	[18]
Anti-cancer	[18,24]
Antioxidant	[22]
Skin-beneficial	[23]

**Table 2:** Different actions of *Chlorella* sp. on human health and related bibliographical references.

*Chlorella* sp. Medicinal Properties	Ref.
Anti-cancer	[25,27,34]
Antioxidant	[27,28,29]
Anti-inflammatory	[30]
Antibacterial, antimicotic	[31,32,33]

**Table 3:** Effects of cyanobacteria and microalgae on oral health: outcomes of the examined in vitro/in vivo studies, potential therapeutic applications, and related bibliographic references.

Species/Source	Type of Study	Results	Potential Application	Ref.
*S. platensis* extract containing Calcium spirulan (Ca-SP)	In vitro	Inhibition of HSV-1, Kaposi sarcoma-associated herpes virus, and human herpes virus 8.	Prophylactic treatment of herpes viruses infections.	[36]
*S. fusiformis*	In vivo	Complete regression of precancerous lesions in 45% of subjects.	Chemopreventive therapy for tobacco-induced oral leucoplakia.	[39]
Astaxanthin	In vitro	Decrease of IL-6 and TNF-α and increase of cell proliferation in oral lichen planus.	Anti-inflammatory treatment for oral lichen planus.	[40]
Astaxanthin	In vivo	Decrease of the incidence of oral pre-neoplastic lesions and cell proliferation activity in rats after 8 weeks of treatment.	Prevention of 4-NQO-induced tongue carcinogenesis.	[41,42]
*S. platensis*	In vivo	Decrease of dysplastic changes on hamsters’ buccal pouch after 14 weeks of treatment.	Oral cancer preventive therapy.	[43,44]
*Spirulina-Dunaliella* extract containing astaxanthin	In vivo	Inhibition of JAK-2/STAT-3 downstream events in hamster buccal pouch tumor progression.	Oral cancer therapy.	[45,46]
GM15 peptide from *Spirulina Arthrospira platensis*	In vitro	Scavenge of superoxide and hydroxyl radicals and reduction of intracellular oxidative stress.	Antioxidant treatment for oral cancer.	[47]
FAME extracted from *S. intermedius*	In vitro	Inhibition of *S. aureus, S. mutans*, *B. cereus, E. coli, P. aeruginosa, A. parasiticus,* and *C. albicans*.	Antimicrobial therapy against Gram-positive bacteria, Gram-negative bacteria, and fungi.	[54]
*C. calcitrans, S. costatum, C. turgidus,* and *N. oceanica*	In vitro	Inhibition of *S. aureus, S. pyogenes*, *B. subtilis, S. costatum,* and *C. turgidus* showed also antimycotic action.	Antibiotics and fungicides.	[55]
Acetone extracts of *O. latevirens*	In vitro	Inhibition *of S. aureus, S. mutans,* and *C. albicans*.	Antibiotic and fungicide.	[56]
Ethanol extracts of *Phormidium corium*	In vitro	Inhibition of *M. mutans* and *S. aureus*.	Antibiotic.	[56]
Extract of *L. martensiana*	In vitro	Inhibition of *B. subtilis, S. aureus, E. coli*.	Antibiotic.	[56]
Extract of *C. minor* and *M. aeruginosa*	In vitro	Inhibition of *C. albicans*.	Fungicides.	[56]
Silver nanoparticles biosynthesized from *S. platensis* extract	In vitro	Inhibition of *S. mutans*, *E. faecalis,* and *S. aureus.*	Antibiotic.	[60]
Locally derived *S. platensis* gel	In vivo	Beneficials in the treatment of chronic periodontitis.	Co-adjuvant in the non-surgical treatment of periodontitis.	[63]
Systemic astaxanthin administration	In vivo	Reduction of alveolar bone loss in ligature-induced periodontitis in rats.	Treatment of periodontitis.	[64]
Cyp, an *Oscillatoria planktothrix FP1*-derived lipopolysaccharide	In vitro	Inhibition of TNF-α, IL-1β, and IL-8 expression.	Treatment of periodontitis.	[67]
Systemic *S. platensis* administration	In vivo	Significant improvement in oral submucous fibrosis symptoms after 3 months of therapy.	Adjuvant therapy in the management of oral submucous fibrosis.	[69]
Systemic *S. platensis* administration in addition to corticosteroid injections	In vivo	Highly significant clinical improvements in oral submucous fibrosis after 3 months of therapy.	Adjuvant therapy in the management of oral submucous fibrosis.	[70]
Astaxanthin	In vivo	Increase of saliva flow after 72 weeks of treatment.	Hyposalivation treatment.	[72]
Astaxanthin	In vitro and in vivo	Increase of saliva flow and decrease of oxidative stress markers.	Hyposalivation treatment.	[73]
*Chlorella*-derived multicomponent supplementation	In vivo	Increase of saliva flow in subjects with lower levels of saliva secretion.	Hyposalivation treatment.	[74]
*C. vulgaris* extract in conjunction with amminosulphurate supplementation	In vivo	Reduction of Hg++, Ag, Sn, and Pb in subjects with long-term titanium dental implants and/or amalgam fillings.	Heavy metal detoxyfing agents.	[75]
Sodium alendronate incorporated into biosilica shells of cultured *Thalassiosira weissflogii* diatoms	In vitro	Decrease of metabolic activity of J774 osteoclast-like cells.	Drug-carrier for bifosphonates.	[76]
